# Predicting Factors Associated with Extended Hospital Stay After Postoperative ICU Admission in Hip Fracture Patients Using Statistical and Machine Learning Methods: A Retrospective Single-Center Study

**DOI:** 10.3390/healthcare13192507

**Published:** 2025-10-02

**Authors:** Volkan Alparslan, Sibel Balcı, Ayetullah Gök, Can Aksu, Burak İnner, Sevim Cesur, Hadi Ufuk Yörükoğlu, Berkay Balcı, Pınar Kartal Köse, Veysel Emre Çelik, Serdar Demiröz, Alparslan Kuş

**Affiliations:** 1 Department of Anaesthesiology and Reanimation, Faculty of Medicine, Kocaeli University, 41001 Kocaeli, Turkeyufukyorukoglu@gmail.com (H.U.Y.); bberkaybalciii@gmail.com (B.B.); pnrkrtl0@gmail.com (P.K.K.); alparslankus@gmail.com (A.K.); 2 Department of Biostatistics and Medical Informatics, Faculty of Medicine, Kocaeli University, 41001 Kocaeli, Turkey; b.s.balci@gmail.com; 3 Department of Orthopedics and Traumatology, Faculty of Medicine, Kocaeli University, 41001 Kocaeli, Turkeyserdardemiroz@hotmail.com (S.D.); 4 Department of Computer Engineering, Kocaeli University, 41001 Kocaeli, Turkey; binner@kocaeli.edu.tr

**Keywords:** hip fracture, postoperative intensive care, hospital length of stay, machine learning, predictive modeling, SHAP analysis, XGBoost

## Abstract

**Background:** Hip fractures are common in the elderly and often require ICU admission post-surgery due to high ASA scores and comorbidities. Length of hospital stay after ICU is a crucial indicator affecting patient recovery, complication rates, and healthcare costs. This study aimed to develop and validate a machine learning-based model to predict the factors associated with extended hospital stay (>7 days from surgery to discharge) in hip fracture patients requiring postoperative ICU care. The findings could help clinicians optimize ICU bed utilization and improve patient management strategies. **Methods:** In this retrospective single-centre cohort study conducted in a tertiary ICU in Turkey (2017–2024), 366 ICU-admitted hip fracture patients were analysed. Conventional statistical analyses were performed using SPSS 29, including Mann–Whitney U and chi-squared tests. To identify independent predictors associated with extended hospital stay, Least Absolute Shrinkage and Selection Operator (LASSO) regression was applied for variable selection, followed by multivariate binary logistic regression analysis. In addition, machine learning models (binary logistic regression, random forest (RF), extreme gradient boosting (XGBoost) and decision tree (DT)) were trained to predict the likelihood of extended hospital stay, defined as the total number of days from the date of surgery until hospital discharge, including both ICU and subsequent ward stay. Model performance was evaluated using AUROC, F1 score, accuracy, precision, recall, and Brier score. SHAP (SHapley Additive exPlanations) values were used to interpret feature contributions in the XGBoost model. **Results:** The XGBoost model showed the best performance, except for precision. The XGBoost model gave an AUROC of 0.80, precision of 0.67, recall of 0.92, F1 score of 0.78, accuracy of 0.71 and Brier score of 0.18. According to SHAP analysis, time from fracture to surgery, hypoalbuminaemia and ASA score were the variables that most affected the length of stay of hospitalisation. **Conclusions:** The developed machine learning model successfully classified hip fracture patients into short and extended hospital stay groups following postoperative intensive care. This classification model has the potential to aid in patient flow management, resource allocation, and clinical decision support. External validation will further strengthen its applicability across different settings.

## 1. Introduction

The world population is aging. Hip fractures, one of the major problems faced by the elderly, result in severe morbidity and mortality [1]. The global prevalence of hip fractures is predicted to rise in line with the population, with annual case numbers being expected to exceed 6 million by 2050 [2,3]. The majority of patients with hip fractures require surgical procedures. Due to the operation itself, comorbidities, and high American Society of Anesthesiologists (ASA) scores, these patients are admitted to the postoperative intensive care unit (ICU). Length of hospital stay following postoperative intensive care is an important determinant of recovery and of both costs and the burden on health services. Prolonged hospitalizations increase complications such as infection, polypharmacy, pressure ulcers, and blood transfusions, while imposing significant costs on the health system [4].

Several factors affect the length of hospital stay including age, comorbidities, the type of fracture, the anesthetic technique employed, postoperative complications, nutritional status, early ambulation, blood transfusion requirements, and compliance with the rehabilitation process [5,6,7,8,9]. Traditional statistical models are frequently applied to analyze this multifactorial process. However, traditional methods exhibit only limited success in predicting lengths of hospital stay due to complex, multivariable, and non-linear relationships [5].

Artificial intelligence (AI), which is rapidly becoming part of daily life, and machine learning (ML) models, a sub-branch thereof, are highly promising in terms of analyzing large datasets and making more accurate predictions [10]. Machine learning is more capable of learning multivariable and non-linear relationships than logistic regression models, can provide individualized predictions, and can be integrated onto clinical decision support systems [11,12,13]. Good ML models, such as Random Forest (RF), Extreme Gradient Boosting (XGBoost), decision trees (DT), and binary logistic regression, are powerful predictive tools for health data analysis [12,14].

Studies involving ML in patients with hip fractures frequently involve the prediction of mortality, delirium, blood transfusion, and postoperative complications [12,14,15,16,17]. These patients are frequently admitted to postoperative intensive care following hip fracture surgery. However, there is a lack of studies on developing ML-based models to identify the factors affecting the length of hospital stay following postoperative intensive care. Such a model may optimize patient care processes, reduce health care costs, and enable the more efficient use of intensive care and hospital bed capacities.

The purpose of this study was to develop and internally validate a prognostic prediction model for extended hospital stay (>7 days from surgery to discharge) in hip fracture patients requiring postoperative ICU care. By comparing conventional regression with multiple machine learning algorithms and applying SHAP analysis for interpretability, we sought to provide a clinically relevant and transparent tool that can support ICU resource allocation and perioperative management. In this way, clinicians will be able to make more effective use of intensive care beds and manage patient care procedures more effectively.

## 2. Method

### 2.1. Study Design

This research was conducted as a retrospective, observational cohort study at the Kocaeli University Medical of Faculty Hospital, Türkiye. Approval was received from the Kocaeli University Medical of Faculty Ethics Committee (approval no. GOKAEK-2024/06.22, protocol no. 2024/180), and the study was registered with ClinicalTrials.gov (registration no. NCT06392048; registration date 17 April 2024). All procedures were carried out in accordance with the Helsinki Declaration and its later amendments. All patients’ data were anonymized. This manuscript was prepared in accordance with the TRIPOD [18] (Transparent Reporting of a multivariable prediction model for Individual Prognosis or Diagnosis) reporting guideline. A completed TRIPOD checklist is available in Supplementary Table S1.

### 2.2. Patient Selection

Patients who underwent surgery due to hip fractures and who were admitted to the postoperative ICU between January 2017 and March 2024 were included in the study.

The inclusion criteria were

- Receipt of surgery due to primary hip fracture, and
- Admission to the postoperative ICU.

Exclusion criteria were

- Deficient basic data records (patients with missing demographic data, operation data, or outcome variables), (none in this cohort)
- Age under 18 (n = 1)
- Receipt of revision surgery (n = 3)

**Figure 1:**
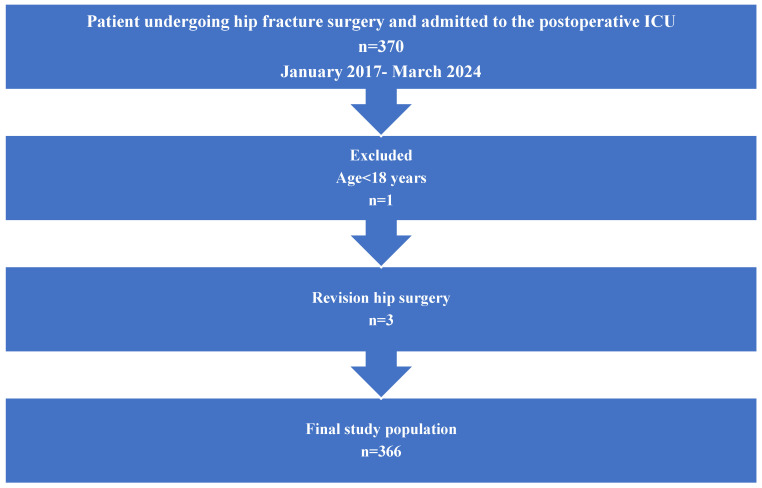
Flowchart of patient selection and exclusions.

There were no patients with missing demographic, operative, or outcome data in this cohort; therefore, no cases were excluded due to incomplete records. After exclusions, the final study population comprised 366 patients. The patient selection process is summarized in Figure 1.

The primary outcome was extended length of stay (LOS). LOS was defined as the total number of days from the date of surgery until hospital discharge, including both ICU and subsequent ward stay. For analytic purposes, LOS was dichotomized at 7 days, with >7 days classified as extended stay.

### 2.3. Patient Characteristics

The patient data were retrieved retrospectively from the hospital information record and management system.

Consistent with the previous literature, risk factors capable of affecting the length of hospital stay in elderly hip fracture patients were evaluated under three headings, preoperative, postoperative, and complication related.

Preoperative variables: age, gender, marital status, previous myocardial infarction, congestive heart failure, peripheral vascular disease, stroke, dementia, chronic obstructive pulmonary disease COPD, peptic ulcer, connective tissue diseases, liver disease, diabetes mellitus, hemiplegia, chronic kidney disease, solid tumor, hematological malignancy (leukemia/lymphoma), polypharmacy (≥5 drugs), fracture type (intertrochanteric/femur neck/subtrochanteric), anesthesia method (spinal/general/combined spinal epidural), ASA score, laboratory data (hemoglobin, platelets, leukocytes, and albumin), time from fracture to surgery (TFS), and the surgical procedure performed (total/partial hip prosthesis/cannulated screw/proximal femoral nail(PFN)/dynamic hip screw- dynamic condylar screw (DHS-DCS).

Postoperative variables: postoperative laboratory values (hemoglobin, platelets, leukocytes, and albumin), postoperative anticoagulant use, length of ICU stay, and total length of hospital stay.

Complications: anemia, heart failure, hypoalbuminemia, electrolyte imbalance, pneumonia, deep vein thrombosis, urinary infection, pulmonary embolism, liver dysfunction, delirium, and mortality.

All available demographic, clinical and laboratory variables were screened for potential predictive value. There were no missing data in this cohort. Continuous laboratory variables were analysed first as continuous measures in univariate analyses and were subsequently dichotomised according to widely accepted clinical reference ranges to enhance interpretability (e.g., WBC < 3460/μL = low; Hgb < 12.1 g/dL = anaemia; Alb < 3.5 g/dL = hypoalbuminaemia; Plt < 172,000/μL = low; CRP > 5 mg/L = elevated). Categorical variables were binary coded (0 = absent; 1 = present). As all predictors were binary or categorised by thresholds, normalisation was not required. Detailed information on data preprocessing and variable definitions is provided in Supplementary Table S2.

### 2.4. Statistical Analysis

Statistical analyses were performed using IBM SPSS version 29.0 software (IBM Corp., Armonk, NY, USA) [19]. The normality assumption was assessed using Kolmogorov–Smirnov’s test. Continuous variables were expressed as median and interquartile range (IQR), since the normality assumption was not met. Categorical variables were expressed as counts (n) and percentages. In this study, we employed a binary classification model to distinguish between short (≤7 days) and extended (>7 days) hospital stays following surgery in hip fracture patients requiring postoperative ICU care. The 7-day threshold was chosen based on the median length of hospital stay in our cohort, providing a clinically meaningful cutoff that reflects typical patient recovery times. Although continuous prediction may offer more granular insights, binary classification simplifies clinical decision-making and risk stratification in real-world settings.

Comparisons between the groups in terms of continuous variables such as age, Charlson comorbidity index (CCI), white blood count (WBC), albumin (Alb), hemoglobin (Hgb), platelet count (Plt), C-reactive protein (CRP), time from fracture to surgery (TFS), and length of ICU stay were performed using the Mann–Whitney U test. Comparisons between groups for categorical variables such as sex, fracture type, marital status, comorbidities, surgical procedures, anesthesia management, perioperative management, and postoperative complications were conducted using the chi-squared test with Bonferroni correction. Independent risk factors for the length of hospital stay were identified using multivariate analysis with binary logistic regression. Due to the large number of parameters capable of affecting the length of hospital stay, Least Absolute Shrinkage and Selection Operator (LASSO) regression analysis was applied to select the most relevant features, reduce overfitting, and cope with multicollinearity before the multivariate analysis. LASSO regression was performed in R (version 4.3.0) using the ‘glmnet’ package. The selected features were TFS, WBC, Hgb, fracture type, peptic ulcer disease (PUD), liver disease, solid tumor, polypharmacy, surgical procedures, anesthesia, ASA, anemia, hypoalbuminemia, electrolyte imbalance, and pneumonia. In order to better assess the effects of the WBC and Hgb on the length of hospital stay, these parameters were classified as low or high based on their reference values, before being included in the multivariate analysis. A *p*-value of <0.05 was considered statistically significant. All steps of data preprocessing and variable definitions, including handling of missing data, variable coding, dichotomization, and feature selection, are summarized in Supplementary Table S3.

### 2.5. Machine Learning Techniques

In addition to statistical methods, ML techniques were applied to identify the features with the most significant effects on length of hospital stay following hip fracture surgery. Feature importance was determined by means of widely used ML techniques, such as binary logistic regression, RF, XGBoost and DT with selected variables. Machine learning techniques were performed in R (version 4.3.0). The packages employed were:

For the binary logistic model: the ‘caret’ package
For the RF model: the ‘randomForest’ package
For the XGBoost model: the ‘xgboost’ package
For the DT model: the ‘rpart’ package

The dataset was split into training (80%) and testing (20%) subsets. Hyperparameter tuning and model performance evaluation were conducted exclusively on the training set using stratified 10-fold cross-validation. For each fold, models were trained on 90% of the training data and validated on the remaining 10%. Hyperparameters were tuned as follows: logistic regression with LASSO regularization (alpha = 1, lambda = 0.1); random forest with mtry values of 2, 4, 6, and 8; XGBoost with 600 rounds, max_depth = 1, learning rate = 0.01, gamma = 0.1, colsample_bytree = 0.9, min_child_weight = 1, and subsample = 0.9; and decision tree tuned over complexity parameter cp from 0.001 to 0.05 in increments of 0.005.

The performance of each model was evaluated using multiple metrics, including accuracy, precision, recall, F1-score, Brier score, and area under the ROC curve (AUROC). AUROCs were plotted for each model for performance comparisons. Pairwise AUROC comparisons were performed by using the DeLong test. The XGBoost model exhibited the best performance, consistently achieving higher performance across multiple evaluation metrics. In order to interpret the results of the XGBoost model, SHAP values were computed and visualized using a summary plot, which displayed the relative importance of each feature.

## 3. Results

The baseline demographic and clinical characteristics of the study population are summarized in Table 1. Table 1 also presents the comparisons between the groups with short hospital stay and extended hospital stay.

The logistic regression analysis revealed several significant factors of the length of hospital stay in patients with hip fractures. Key findings from both univariable and multivariable analyses are summarized in Table 2.

The full set of regression coefficients, including intercept and standard errors for the final logistic regression model, is presented in Supplementary Table S4 to facilitate reproducibility and potential external validation.

**Figure 2:**
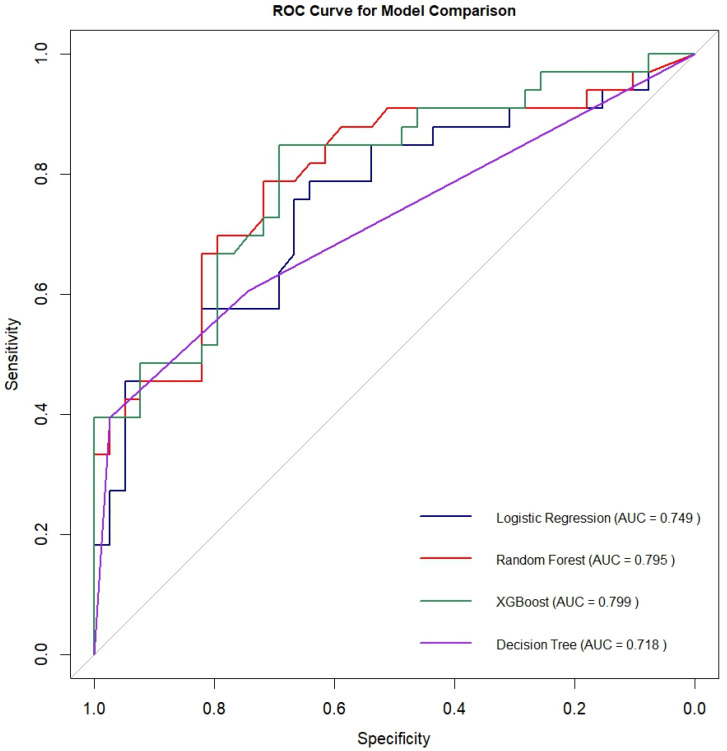
The AUROC values for the models. The diagonal line in the ROC curve represents random prediction and serves as a baseline.

The performance metrics for all models are presented in Table 3. The AUROC values for the models are illustrated in Figure 2. Among the models, XGBoost demonstrated the best performance, except for precision. The XGBoost model gave an AUROC of 0.80, precision of 0.67, recall of 0.92, F1 score of 0.78, accuracy of 0.71 and Brier score of 0.18. Pairwise AUROC comparisons using the DeLong test showed that XGBoost differed significantly from Logistic Regression (*p* = 0.021) and Decision Tree (*p* = 0.039). No other differences were statistically significant (*p* > 0.05).

**Figure 3:**
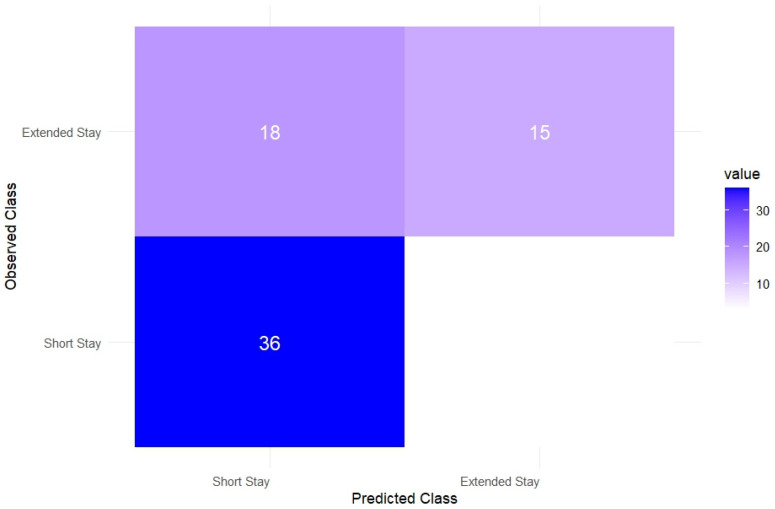
Confusion matrix for the XGBoost model predicting extended hospital stay.

The confusion matrix for the best-performing model is presented in Figure 3.

**Figure 4:**
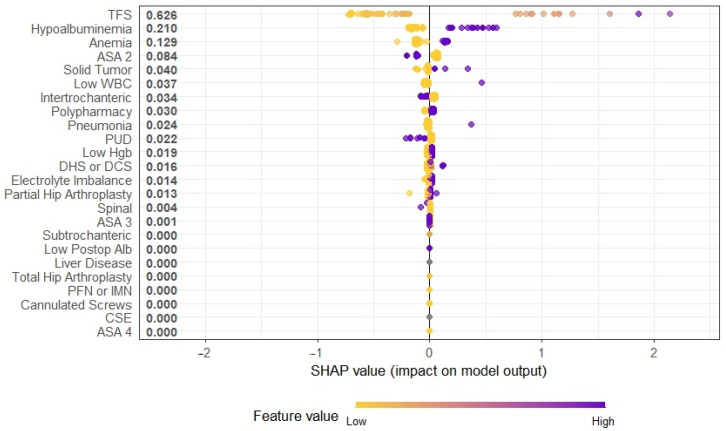
SHAP values of the XGBoost model. TFS: Time from Fracture to Surgery, WBC: White blood count, Hgb: Hemoglobin, PUD: Peptic Ulcer Disease, ASA: American Society of Anesthesiologists, DHS: Dynamic Hip Screw, DCS: Dynamic Compression Screw, PFN: Proximal Femoral Nail, IMN: Intramedullary Nail, CSE: Combined spinal epidural, Alb: Albumin.

SHAP scores were used to visualize feature effects on the model output, as shown in Figure 4. The SHAP values on the horizontal axis indicate the magnitude and direction of the effect, while the vertical axis lists the features affecting model predictions. The color gradient from yellow to purple represents actual attribute values, ranging from lower to higher magnitudes.

## 4. Discussion

In this study, we used a complete and balanced dataset, with men and women each representing 50% of the cohort, and 32.2% of patients aged over 80. This demographic distribution aligns with the typical profile of hip fracture patients admitted to the postoperative ICU.

Our objective was twofold: first, to compare traditional statistical methods and commonly used ML algorithms in identifying the factors associated with extended hospital stay in patients monitored in the postoperative ICU following hip fracture surgery; and second, develop an interpretable predictive model capable of being integrated into clinical practice. The models were therefore trained to classify lengths of stay as either “short” or “extended” based on the median length of hospital stay following hip fracture, which was 7 days. Although this threshold corresponded to the median LOS in our cohort, it also has clinical relevance. International guidelines emphasize that most patients should recommence their activities of daily living (re-enablement) within 2–5 days after hip fracture surgery; delays beyond this period are frequently related to complications such as pain, hypotension, bowel or bladder dysfunction, or delirium [20]. Therefore, a hospital stay exceeding 7 days can be considered a clinically meaningful deviation from expected recovery trajectories. While local LOS distributions may differ across institutions, we believe that this cut-off provides a pragmatic and interpretable outcome measure for prediction modeling. Although continuous prediction may offer more granular insights, binary classification simplifies clinical decision-making and risk stratification in real-world settings.

The LASSO regression method was used for variable selection, and the most significant clinical characteristics for inclusion in the models were identified. The XGBoost model exhibited the best performance among the four distinct ML algorithms applied (accuracy: 0.71, precision: 0.67, and recall: 0.92). The AUROC score was 0.80 and the Brier score 0.18. These metrics show that the model exhibited good predictive accuracy and a powerful ability to distinguish between the classes.

Variables identified as significant at regression analysis to a large extent coincided with the characteristics revealed by the SHAP analysis applied with the ML models. According to the SHAP analysis, the variable with the greatest effect on the model prediction was TFS. This finding suggests that the development of complications in the postoperative period may result in delayed mobilization and a prolongation of care. It is also consistent with modern hip fracture management guidelines that recommend early surgery [20]. The contribution of postoperative hypoalbuminemia and anemia to high SHAP suggests that dietary state and haematological adequacy predict hospital stay. Anaemia and hypoalbuminaemia were significant in univariate analysis but not independent predictors after multivariable adjustment, perhaps due to surgical complications. While clinically relevant, their prognostic effect may be modulated by other variables. Our balanced dataset and consistent perioperative methods strengthen the internal validity of these findings from a single-center cohort. Since each institution has its own patient demographics and therapeutic pathways, external validation in varied settings is necessary to adjust the model to individual decision-making and resource optimization. The significant contribution of an ASA score of 2 suggests that moderately comorbid patients may have more varied clinical outcomes. However, the limited but measurable effects on the intertrochanteric fracture model and surgical treatments like DHS/DCS suggest that anatomical fracture types and fixation methods may indirectly aid recovery.

The close-to-zero SHAP coefficients of variables such as liver disease, connective tissue diseases, total hip prosthesis, and some fixation techniques (e.g., proximal femoral nail, intramedullary nail, combined spinal epidural, and cannulated screws) show that these variables have no significant impact on the model output. This emphasizes that variables in clinical decision support systems should be selected not solely on the basis of their importance in the literature but also of their data-based contributions.

Beyond statistical significance, our findings also carry important clinical implications. Extended hospital stays (>7 days) increased postoperative complications, including pneumonia and hypoalbuminemia, and ICU and ward use. Although no cost-effectiveness analysis was performed, these findings suggest that early identification of patients at risk for extended hospitalization could enable targeted perioperative measures including nutritional optimization and early mobilization. Such approaches may reduce complications, shorten hospitalization, and enhance patient outcomes while improving healthcare resource consumption.

Machine learning methods are frequently employed for predicting clinical outcomes such as mortality [12], delirium [15], and functional improvement [3] in hip fracture cases. A model developed by Kitcharanant et al. [12] showed that among various algorithms for predicting one-year mortality following fragile hip fracture, the Random Forest model exhibited the greatest success, with 95% accuracy and a high AUC (0.99). Delirium is one of the most frequent postoperative complications in geriatric hip fracture patients and may contribute to extended hospitalization. However, even advanced monitoring strategies, such as automated EEG-based delirium detection, failed to shorten length of stay in a large multicentre randomized controlled trial. These findings, together with our results, support the view that hospital stay in hip fracture patients is determined by multifactorial influences [21].

Zhao et al. [15] achieved accuracy rates as high as 87% using algorithms such as XGBoost and SVM based on clinical and anesthetic data for predicting the risk of postoperative delirium. Similarly, Lin et al.’s [3] study emphasized the power of clinically significant variables to predict functional improvement in elderly hip fracture patients using the Random Forest algorithm together with SHAP analysis.

Interestingly, during the COVID-19 pandemic, a prospective cohort study [22] from Taiwan found no significant difference in hospital length of stay between patients with and without COVID-19 pneumonia, likely reflecting accelerated discharge policies. Nevertheless, functional outcomes were significantly difference in the infected group. By contrast, our study identified postoperative pneumonia as an independent predictor of a prolonged hospital stay (adjusted odds ratio (aOR) 3.28, *p* = 0.038).

Machine learning methods are becoming increasingly commonly employed to predict different clinical outcomes in hip fracture cases. Zhu et al. [14] compared eight different ML algorithms in a model they developed to predict postoperative blood transfusion requirements in patients receiving total hip prosthesis due to femur neck fracture. Those authors reported that the highest accuracy (95%) was achieved with logistic regression. Similarly, Lin et al. [13] reported that artificial neural network models yielded higher AUC scores that logistic regression in predicting one-year mortality in elderly hip fracture patients and that these possess the potential to be integrated into clinical decision support systems.

As this was a single-center study conducted in a tertiary ICU in Turkey and limited to postoperative ICU-admitted patients, the generalizability of the findings is restricted, underscoring the need for external multicenter validation. Nevertheless, the relatively balanced dataset in our cohort strengthens the internal validity of the results and provides a solid basis for future multicenter studies.

This study primarily focused on model development and internal validation; the proposed tool could in principle be integrated into a web-based risk calculator or embedded within ICU clinical information systems to provide real-time predictions. Such an approach may support clinicians in identifying high-risk patients earlier and optimizing perioperative resource allocation. Nevertheless, before any clinical implementation, external multicenter validation would be valuable to further strengthen the robustness and generalizability of the model.

In contrast to those studies, the present research describes a novel ML model for predicting an insufficiently investigated outcome, extended hospital stays in patients with hip fracture admitted to the postoperative ICU. Additionally, the combined use of methods such as LASSO and SHAP that provide variable selection and model explainability enhanced both the clinical significance and practicability of the model. While previous studies have generally focused on the prediction of mortality or complications, the present study sought to produce an output with a direct effect on resource planning, the effective use of intensive care and hospital beds, and patient management.

This study has several limitations. First, the retrospective single-center design limits the generalizability of the results. Second, some clinically relevant variables that may affect postoperative recovery—such as functional status, frailty index, cognitive impairment, or rehabilitation compliance—were not included due to data unavailability. Third, the definition of extended hospital stay was based on the median length of stay (7 days) in this cohort, which may limit external applicability in hospitals with different care protocols. Finally, external validation in independent datasets is necessary to assess the model’s performance and utility across different healthcare settings. Nonetheless, the balanced nature of the dataset and standardized perioperative care contribute to the internal validity of our findings.

## 5. Conclusions

The ML model developed in this study to identify the factors associated with extended hospital stay in patients admitted to the postoperative ICU following hip fracture has the potential for clinical use due to its high accuracy and explainability. The combined use of LASSO regression and SHAP analyses enhanced the model’s predictivity and interpretability. Time from fracture to surgery following fracture emerged as the most important determinant.

Compared to the frequently examined variables of mortality, complications, and transfusion risk in the existing literature, this model focusing on extended hospital stay will fill a significant gap in terms of optimizing clinic management and more effective use of health resources. The external validation of this model and its integration into decision support systems can be strengthened in the future with data from different centers.

## Figures and Tables

**Table 1:** Comparison of baseline demographic and clinical characteristics between short and extended hospital stay groups.

	Total (n = 366)	Short Hospital Stay (n = 198)	Extended Hospital Stay (n = 168)	*p*
Demographic and Baseline Characteristics				
**Age, median (IQR)**	73.5 (60.7–82.0)	73.0 (59.0–81.0)	75.0 (63.2–82.0)	0.373
**Age Distribution, n (%)**				0.493
<50 years	54 (14.8)	29 (14.6)	25 (14.9)	
50–59 years	33 (9.0)	22 (11.1)	11 (6.5)	
60–69 years	62 (16.9)	30 (15.2)	32 (19.0)	
70–79 years	99 (27.0)	56 (28.3)	43 (25.6)	
≥80 years	118 (32.2)	61 (30.8)	57 (33.9)	
**Sex, n (%)**				0.074
Female	183 (50.0)	108 (54.5)	75 (44.6)	
Male	183 (50.0)			
**Marital Status, n (%)**				0.205
Married	286 (78.1)	160 (80.8)	126 (75.0)	
Single	80 (21.9)	38 (19.2)	42 (25.0)	
**CCI, median (IQR)**	0 (0–2)	0 (0–2)	0 (0–3)	**0.003**
**WBC (µL), median (IQR)**	9000 (7000–11,600)	9450 (7400–11,925)	8250 (6300–11,225)	**0.002**
**Alb (g/L), median (IQR)**	36.8 (32–40)	38 (34.5–41)	34.9 (31–38.9)	**<0.001**
**Hgb (g/dL), median (IQR)**	11.5 (10.2–13)	12 (10.5–13.2)	11 (10–12.6)	**<0.001**
**Plt (µL), median (IQR)**	209,000 (171,000–271,000)	207,000 (170,500–266,000)	214,500 (173,500–274,000)	0.787
**Postop WBC (µL), median (IQR)**	10,300 (8000–13,000)	10,400 (8500–13,325)	9950 (7500–12,700)	**0.035**
**Postop Alb (g/L), median (IQR)**	29 (25–32)	30 (27–33)	27 (24–30)	**<0.001**
**Postop Hgb (g/dL), median (IQR)**	10.1 (9.3–11)	10.3 (9.5–11.2)	9.7 (9–10.7)	**<0.001**
**Postop Plt (µL), median (IQR)**	183,000 (147,000–243,750)	182,000 (148,750–246,250)	184,000 (136,250–233,250)	0.574
**CRP (mg/L), median (IQR)**	96.6 (38.7–152.2)	89.6 (34.8–143.2)	103 (51.4–158.6)	**0.049**
**TFS, median (IQR)**	2 (1–3)	1 (1–2)	3 (1–5)	**<0.001**
**Length of ICU Stay, median (IQR)**	1 (1–1)	1 (1–1)	1 (1–1)	**<0.001**
**Fracture Type, n (%)**				**0.019**
Femoral Neck	207 (56.6)	101 (51) ^a^	106 (63.1) ^b^	
Intertrochanteric	104 (28.4)	68 (34.3) ^a^	36 (21.4) ^b^	
Subtrochanteric	55 (15)	29 (14.6) ^a^	26 (15.5) ^a^	
**Comorbidities**				
**Previous MI, n (%)**	56 (15.3)	26 (13.1)	30 (17.9)	0.244
**CHF, n (%)**	69 (18.9)	33 (16.7)	36 (21.4)	0.284
**PAH, n (%)**	9 (2.5)	5 (2.5)	4 (2.4)	1.0
**CVD, n (%)**	44 (12)	24 (12.1)	20 (11.9)	1.0
**Dementia, n (%)**	38 (10.4)	21 (10.6)	17 (10.1)	1.0
**COPD, n (%)**	28 (7.7)	12 (6.1)	16 (9.5)	0.296
**PUD, n (%)**	27 (7.4)	12 (6.1)	15 (8.9)	0.398
**Connective Tissue Disease, n (%)**	6 (1.6)	2 (1)	4 (2.4)	NA
**Liver Disease, n (%)**	6 (1.6)	4 (2)	2 (1.2)	0.691
**DM, n (%)**	100 (27.3)	48 (24.2)	52 (31)	0.159
**Hemiplegia, n (%)**	7 (1.9)	7 (3.5)	0 (0)	**0.017**
**CKD, n (%)**	68 (18.6)	26 (13.1)	42 (25)	**0.004**
**Solid Tumor, n (%)**	49 (13.4)	17 (8.6)	32 (19)	**0.006**
**Leukemia, n (%)**	6 (1.6)	3 (1.5)	3 (1.8)	1.0
**Lymphoma, n (%)**	2 (0.5)	0 (0)	2 (1.2)	NA
**Polypharmacy, n (%)**	230 (62.8)	112 (56.6)	118 (70.2)	**0.009**
**Surgical Procedures**				
**Surgical Procedures, n (%)**				**0.005**
Partial Hip Arthroplasty	176 (48.1)	80 (40.4) ^a^	96 (57.1) ^b^	
Total Hip Arthroplasty	14 (3.8)	6 (3) ^a^	8 (4.8) ^a^	
DHS or DCS	111 (30.3)	74 (37.4) ^a^	37 (22) ^b^	
PFN or IMN	40 (10.9)	25 (12.6) ^a^	15 (8.9) ^a^	
Cannulated Screws	25 (6.8)	13 (6.6) ^a^	12 (7.1) ^a^	
**Anesthetic Management**				
**Anesthesia, n (%)**				0.103
General	338 (92.3)	178 (89.9)	160 (95.2)	
Spinal	18 (4.9)	14 (7.1)	4 (2.4)	
Combined spinal epidural	10 (2.7)	6 (3)	4 (2.4)	
**Perioperative Management**				
**Blood Transfusion, n (%)**	143 (39.1)	66 (33.3)	77 (45.8)	**0.018**
**ASA, n (%)**				**<0.001**
1	9 (2.5)	5 (2.5) ^a^	4 (2.4) ^a^	
2	121 (33.1)	84 (42.4) ^a^	37 (22) ^b^	
3	195 (53.3)	92 (46.5) ^a^	103 (61.3) ^b^	
4	41 (11.2)	17 (8.6) ^a^	24 (14.3) ^a^	
**PCA Use, n (%)**	26 (7.1)	17 (8.6)	9 (5.4)	0.320
**Postop Anticoagulation Usage, n (%)**	352 (96.2)	188 (94.9)	164 (97.6)	0.292
**Postoperative Complications**				
**Anemia, n (%)**	157 (42.9)	67 (33.8)	90 (53.6)	**<0.001**
**Heart Failure, n (%)**	4 (1.1)	1 (0.5)	3 (1.8)	NA
**Hypoalbuminemia, n (%)**	101 (27.6)	35 (17.7)	66 (39.3)	**<0.001**
**Electrolyte Imbalance, n (%)**	103 (28.1)	35 (17.7)	68 (40.5)	**<0.001**
**Pneumonia, n (%)**	22 (6)	7 (3.5)	15 (8.9)	0.052
**Deep Vein Thrombosis, n (%)**	2 (0.5)	1 (0.5)	1 (0.6)	NA
**Urinary Tract Infection, n (%)**	1 (0.3)	0 (0)	1 (0.6)	NA
**Pulmonary Embolism, n (%)**	7 (1.9)	2 (1)	5 (3)	0.255
**Hepatic Dysfunction, n (%)**	6 (1.6)	1 (0.5)	5 (3)	0.098
**Delirium, n (%)**	42 (11.5)	25 (12.6)	17 (10.1)	0.558
**In-hospital Mortality, n (%)**	85 (23.2)	40 (20.2)	45 (26.8)	0.172

CCI: Charlson Comorbidity Index, WBC: White blood count, Alb: Albumin, ASA: American Society of Anesthesiologists, Hgb: Hemoglobin, Plt: Platelet count, CRP: C-reactive protein, TFS: Time from Fracture to Surgery, ICU: Intensive Care Unit, MI: Myocardial Infarction, CHF: Congestive Heart Failure, PAH: Pulmonary Arterial Hypertension, CVD: Cerebrovascular Disease, COPD: Chronic Obstructive Pulmonary Disease, DM: Diabetes Mellitus, PUD: Peptic Ulcer Disease, CKD: Chronic Kidney Disease, DHS: Dynamic Hip Screw, DCS: Dynamic Compression Screw, PFN: Proximal Femoral Nail, IMN: Intramedullary Nail, IQR: Interquartile range, NA: Not applicable. Mann–Whitney U test and chi-squared test (with Bonferroni correction) were used for the parameters presented with median (IQR), and n (%), respectively. Different lowercase letters in rows indicate statistically significant differences. Although the median ICU stay was 1 day in both groups, the distributions differed due to a few patients with prolonged stays (range 1–6 vs. 1–48 days), which explains the significant (*p* < 0.001). Although the median CCI was 0 in both groups, the distributions differed due to patients with higher comorbidity scores (range 0–9 vs. 0–14), which explains the significant *p*-value (*p* = 0.003). Statistically significant results (*p* < 0.05) are shown in bold.

**Table 2:** Univariate and multivariate analysis of factors associated with extended hospital stay.

	Univariate Analysis		Multivariate Analysis	
	OR (95% CI)	*p*	aOR (95% CI)	*p*
**TFS**	1.73 (1.48–2.01)	**<0.001**	1.73 (1.46–2.05)	**<0.001**
**WBC (µL)**				
Low	3.40 (1.06–10.88)	**0.039**	5.95 (1.46–24.22)	**0.013**
High (R)	1.0	-	1.0	-
**Hgb (g/dL)**				
Low	2.31 (1.50–3.56)	**<0.001**	1.04 (0.57–1.89)	0.891
High (R)	1.0	-	1.0	-
**Fracture Type**				
Femoral Neck (R)	1.0	**0.022**	1.0	0.510
Intertrochanteric	0.50 (0.31–0.82)	**0.006**	0.97 (0.37–2.49)	0.944
Subtrochanteric	0.85 (0.47–1.55)	0.604	1.62 (0.49–5.38)	0.432
**PUD**				
Present	1.52 (0.69–3.34)	0.298	0.35 (0.11–1.08)	0.068
Absent (R)	1.0	-	1.0	-
**Liver Disease**				
Present	0.58 (0.11–3.23)	0.538	0.16 (0.01–2.66)	0.203
Absent (R)	1.0	-	1.0	-
**Solid Tumor**				
Present	2.51 (1.34–4.70)	**0.004**	1.27 (0.57–2.84)	0.559
Absent (R)	1.0	-	1.0	-
**Polypharmacy**				
Present	1.81 (1.17–2.80)	**0.007**	1.39 (0.70–2.75)	0.343
Absent (R)	1.0	-	1.0	-
**Surgical Procedures**				
Partial Hip Arthroplasty (R)	1.0	-	1.0	-
Total Hip Arthroplasty	1.11 (0.37–3.34)	0.851	1.20 (0.22–6.6)	0.834
DHS or DCS	0.42 (0.25–0.68)	**0.001**	0.66 (0.26–1.71)	0.395
PFN or IMN	0.50 (0.25–1.01)	0.054	0.48 (0.15–1.50)	0.207
Cannulated Screws	0.77 (0.33–1.78)	0.540	2.95 (0.96–9.08)	0.059
**Anesthesia**				
General (R)	1.0	-	1.0	-
Spinal	0.32 (0.10–0.99)	**0.047**	0.48 (0.11–2.12)	0.335
Combined spinal epidural	0.74 (0.21–2.68)	0.648	1.41 (0.31–6.43)	0.656
**ASA**				
1 (R)	1.0	-	1.0	-
2	0.55 (0.14–2.17)	0.393	0.38 (0.07–2.05)	0.259
3	1.40 (0.36–5.37)	0.624	0.63 (0.11–3.46)	0.594
4	1.76 (0.41–7.55)	0.444	0.75 (0.12–4.79)	0.762
**Anemia**				
Present	2.26 (1.48–3.44)	**<0.001**	1.59 (0.85–2.98)	0.145
Absent (R)	1.0	-	1.0	-
**Hypoalbuminemia**				
Present	3.01 (1.87–4.86)	**<0.001**	1.40 (0.69–2.83)	0.347
Absent (R)	1.0	-	1.0	-
**Electrolyte Imbalance**				
Present	3.17 (1.96–5.11)	**<0.001**	1.94 (0.92–4.07)	0.081
Absent (R)	1.0	-	1.0	-
**Pneumonia**				
Present	2.68 (1.06–6.73)	**0.036**	3.28 (1.07–10.09)	**0.038**
Absent (R)	1.0	-	1.0	-

TFS: Time from Fracture to Surgery, WBC: White blood count, Hgb: Hemoglobin, PUD: Peptic Ulcer Disease, ASA: American Society of Anesthesiologists, DHS: Dynamic Hip Screw, DCS: Dynamic Compression Screw, PFN: Proximal Femoral Nail, IMN: Intramedullary Nail. OR: Odds ratio, aOR: Adjusted odds ratio, CI: Confidence interval, R: Reference category. Statistically significant results (*p* < 0.05) were shown in bold.

**Table 3:** Performance metrics of all models calculated on the independent 20% test set.

	AUCROC	Precision	Recall	F1 Score	Accuracy	Brier Score
Logistic Regression	0.75	0.65	0.82	0.73	0.67	0.21
Random Forest	0.79	0.64	0.82	0.72	0.65	0.19
XGBoost	**0.80**	0.67	**0.92**	**0.78**	**0.71**	**0.18**
Decision Tree	0.72	**0.69**	0.74	0.72	0.68	0.19

The best performance for each metric is highlighted in bold.

## Data Availability

The data presented in this study are available on request from the corresponding author. The data are not publicly available due to privacy and ethical restrictions.

## Supplementary Materials

The following supporting information can be downloaded at: https://www.mdpi.com/article/10.3390/healthcare13192507/s1, Table S1: Tripod checklist; Table S2: Patient details and baseline variables; Table S3: Data preprocessing and variable definitions; Table S4: Full coefficients of the multivariate logistic regression model for predicting extended hospital stay.

## References

[1] Veronese N., Maggi S. (2018). Epidemiology and social costs of hip fracture. Injury.

[2] Arinzon Z., Shabat S., Peisakh A., Gepstein R., Berner Y.N. (2010). Gender differences influence the outcome of geriatric rehabilitation following hip fracture. Arch. Gerontol. Geriatr..

[3] Lin C., Liang Z., Liu J., Sun W. (2023). A machine learning-based prediction model pre-operatively for functional recovery after 1-year of hip fracture surgery in older people. Front. Surg..

[4] Leal J., Gray A.M., Prieto-Alhambra D., Arden N.K., Cooper C., Javaid M.K., Judge A., REFReSH Study Group (2016). Impact of hip fracture on hospital care costs: A population-based study. Osteoporos. Int. J. Establ. Result Coop. Eur. Found. Osteoporos. Natl. Osteoporos. Found. USA.

[5] Thornburgh Z., Samuel D. (2022). Factors Influencing Length of Stay and Discharge Destination of Patients with Hip Fracture Rehabilitating in a Private Care Setting. Geriatrics.

[6] Vasu B.K., Ramamurthi K.P., Rajan S., George M. (2018). Geriatric Patients with Hip Fracture: Frailty and Other Risk Factors Affecting the Outcome. Anesth. Essays Res..

[7] Kim C.-H., Lee B.-R., Park J.-S., Kim J.-B., Kwon S.-W., Kim W.-J., Jung K.-J., Jang B.-W., Hong C.-H. (2024). Efficacy of Postoperative Oral Nutritional Supplements in Geriatric Hip Fracture Patients Undergoing Total Hip Arthroplasty. JCM.

[8] Amari T., Matta D., Makita Y., Fukuda K., Miyasaka H., Kimura M., Sakamoto Y., Shimo S., Yamaguchi K. (2023). Early Ambulation Shortened the Length of Hospital Stay in ICU Patients after Abdominal Surgery. Clin. Pract..

[9] Krakers S., Woudsma S., Van Dartel D., Vermeer M., Vollenbroek-Hutten M., Hegeman J., on behalf of the Up&Go After a Hip Fracture Group (2024). Rehabilitation of Frail Older Adults after Hip Fracture Surgery: Predictors for the Length of Geriatric Rehabilitation Stay at a Skilled Nursing Home. JCM.

[10] Shin D. (2023). Algorithms, Humans, and Interactions: How Do Algorithms Interact with People? Designing Meaningful AI Experiences.

[11] Sun H., Wu S., Li S., Jiang X. (2023). Which model is better in predicting the survival of laryngeal squamous cell carcinoma?: Comparison of the random survival forest based on machine learning algorithms to Cox regression: Analyses based on SEER database. Medicine.

[12] Kitcharanant N., Chotiyarnwong P., Tanphiriyakun T., Vanitcharoenkul E., Mahaisavariya C., Boonyaprapa W., Unnanuntana A. (2022). Development and internal validation of a machine-learning-developed model for predicting 1-year mortality after fragility hip fracture. BMC Geriatr..

[13] Lin C.-C., Ou Y.-K., Chen S.-H., Liu Y.-C., Lin J. (2010). Comparison of artificial neural network and logistic regression models for predicting mortality in elderly patients with hip fracture. Injury.

[14] Zhu J., Xu C., Jiang Y., Zhu J., Tu M., Yan X., Shen Z., Lou Z. (2024). Development and Validation of a Machine Learning Algorithm to Predict the Risk of Blood Transfusion after Total Hip Replacement in Patients with Femoral Neck Fractures: A Multicenter Retrospective Cohort Study. Orthop. Surg..

[15] Zhao H., You J., Peng Y., Feng Y. (2021). Machine Learning Algorithm Using Electronic Chart-Derived Data to Predict Delirium After Elderly Hip Fracture Surgeries: A Retrospective Case-Control Study. Front. Surg..

[16] Shi L., Wang X.C., Wang Y.S. (2013). Artificial neural network models for predicting 1-year mortality in elderly pa-tients with intertrochanteric fractures in China. Braz. J. Med. Biol. Res..

[17] DeBaun M.R., Chavez G., Fithian A., Oladeji K., Van Rysselberghe N., Goodnough L.H., Bishop J.A., Gardner M.J. (2021). Artificial Neural Networks Predict 30-Day Mortality After Hip Fracture: Insights From Machine Learning. J. Am. Acad. Orthop. Surg..

[18] Collins G.S., Reitsma J.B., Altman D.G., Moons K.G. (2015). Transparent Reporting of a Multivariable Prediction Model for Individual Prognosis or Diagnosis (TRIPOD): The TRIPOD Statement. Circulation.

[19] IBM Corp (2022). IBM SPSS Statistics for Windows.

[20] Griffiths R., Babu S., Dixon P., Freeman N., Hurford D., Kelleher E., Moppett I., Ray D., Sahota O., Shields M. (2021). Guideline for the management of hip fractures 2020: Guideline by the Association of Anaesthetists. Anaesthesia.

[21] De Fraiture E.J., Schuijt H.J., Menninga M., Koevoets I.A.I., Verheul T.F.M., Van Goor C.W., Nijdam T.M.P., Van Dartel D., Hegeman J.H., Van Der Velde D. (2024). Automated EEG-Based Brainwave Analysis for the Detection of Postoperative Delirium Does Not Result in a Shorter Length of Stay in Geriatric Hip Fracture Pa-tients: A Multicentre Randomized Controlled Trial. JCM.

[22] Tay H.-Y., Wu W.-T., Peng C.-H., Liu K.-L., Yu T.-C., Chen I.-H., Yao T.-K., Chang C.-M., Chua J.-Y., Wang J.-H. (2023). COVID-19 Infection Was Associated with the Functional Outcomes of Hip Fracture among Older Adults during the COVID-19 Pandemic Apex. Medicina.

