# Supplementary material for: Predicting Factors Associated with Extended Hospital Stay After Postoperative ICU Admission in Hip Fracture Patients Using Statistical and Machine Learning Methods: A Retrospective Single-Center Study
Source: Healthcare (Basel). 2025 Oct 2;13(19):2507. doi: 10.3390/healthcare13192507 (PMC12524269; doi:10.3390/healthcare13192507)
Supplement: Supplementary file 1 [file healthcare-13-02507-s001.zip › healthcare-3858287-supplementary.pdf]

Table S1. Tripod checklist

ı		Checklist Item	Page
**Title and abstract**			
Title	1	Identify the study as developing and/or validating a multivariable prediction model, the target population, and the outcome to be predicted.	Title, p.1
Abstract	2	Provide a summary of objectives, study design, setting, participants, sample size, predictors, outcome, statistical analysis, results, and conclusions.	Abstract, p.2
**Introduction**			
Background and objectives	3a	Explain the medical context (including whether diagnostic or prognostic) and rationale for developing or validating the multivariable prediction model, including references to existing models.	Introduction, p.3, para.1–3
	3b	Specify the objectives, including whether the study describes the development or validation of the model or both.	Introduction, p.3, para.4
**Methods**			
Source of data	4a	Describe the study design or source of data (e.g., randomized trial, cohort, or registry data), separately for the development and validation data sets, if applicable.	Methods, Patient Selection, p.4, para.1
	4b	Specify the key study dates, including start of accrual; end of accrual; and, if applicable, end of follow-up.	Methods, Patient Selection, p.4, para.1 (2017–2024)
Participants	5a	Specify key elements of the study setting (e.g., primary care, secondary care, general population) including number and location of centres.	Methods, Patient Selection, p.4, tertiary ICU, Turkey
	5b	Describe eligibility criteria for participants.	Methods, Patient Selection, p.4
	5c	Give details of treatments received, if relevant.	Not applicable (retrospective observational, no interventions specified)
Outcome	6a	Clearly define the outcome that is predicted by the prediction model, including how and when assessed.	Methods, Outcomes, p.5
	6b	Report any actions to blind assessment of the outcome to be predicted.	Not applicable (objective LOS endpoint)
Predictors	7a	Clearly define all predictors used in developing or validating the multivariable prediction model, including how and when they were measured.	Methods, Patient characteristics, p.5–6
	7b	Report any actions to blind assessment of predictors for the outcome and other predictors.	Not applicable (retrospective, EMR extraction)
Sample size	8	Explain how the study size was arrived at.	Methods, Patient Selection, p.4 (final n=366 after exclusions)
Missing data	9	Describe how missing data were handled (e.g., complete-case analysis, single imputation, multiple imputation) with details of any imputation method.	Methods, Patient characteristics, p.5 (no missing values)
Statistical analysis methods	10a	Describe how predictors were handled in the analyses.	Methods, Variable preprocessing, p.6–7
	10b	Specify type of model, all model-building procedures (including any predictor selection), and method for internal validation.	Methods, Modeling, p.6–7(LASSO, ML models, 10-fold CV, 80/20 split)
	10d	Specify all measures used to assess model performance and, if relevant, to compare multiple models.	Methods, Modeling, p.6 (AUROC, accuracy, recall, F1, Brier, HL test)
Risk groups	11	Provide details on how risk groups were created, if done.	Not applicable (binary outcome only)
**Results**			

Participants	13a	Describe the flow of participants through the study, including the number of participants with and without the outcome and, if applicable, a summary of the follow-up time. A diagram may be helpful.	Results, p.4 + Figure 1 (flowchart)
	13b	Describe the characteristics of the participants (basic demographics, clinical features, available predictors), including the number of participants with missing data for predictors and outcome.	Results, Table 1, p.7-9
Model development	14a	Specify the number of participants and outcome events in each analysis.	Results, p.9-10 (168 extended stay events)
	14b	If done, report the unadjusted association between each candidate predictor and outcome.	Results, Table 2, p.9-10
Model specification	15a	Present the full prediction model to allow predictions for individuals (i.e., all regression coefficients, and model intercept or baseline survival at a given time point).	Full logistic regression coefficients including intercept are provided in Supplementary Table S4.
	15b	Explain how to the use the prediction model.	Discussion, p.14 (potential for clinical decision support, SHAP interpretation)
Model performance	16	Report performance measures (with CIs) for the prediction model.	Results, Table 3 + p.10-11(AUROC, precision, recall, F1, Brier)
**Discussion**			
Limitations	18	Discuss any limitations of the study (such as nonrepresentative sample, few events per predictor, missing data).	Discussion, p.14–15
Interpretation	19b	Give an overall interpretation of the results, considering objectives, limitations, and results from similar studies, and other relevant evidence.	Discussion, p.14–15
Implications	20	Discuss the potential clinical use of the model and implications for future research.	Discussion, p.15
**Other information**			
Supplementary information	21	Provide information about the availability of supplementary resources, such as study protocol, Web calculator, and data sets.	Supplementary Materials, Table S1 + note on data availability
Funding	22	Give the source of funding and the role of the funders for the present study.	Funding statement, p.16

Supplementary Table S2. Patient details and baseline variables

Category	Variable	Definition / Coding
**Demographics**	Age	Continuous (years)
	Sex	Male = 0, Female = 1
	Marital status	Married / Single / Widowed
**Comorbidities**	Hypertension	0 = absent, 1 = present
	Diabetes mellitus	0 = absent, 1 = present
	Coronary artery disease / Previous MI	0 = absent, 1 = present
	Congestive heart failure	0 = absent, 1 = present

	Chronic kidney disease	0 = absent, 1 = present
	COPD	0 = absent, 1 = present
	Peripheral vascular disease	0 = absent, 1 = present
	Stroke / Dementia / Hemiplegia	0 = absent, 1 = present
	Peptic ulcer disease (PUD)	0 = absent, 1 = present
	Connective tissue diseases	0 = absent, 1 = present
	Liver disease	0 = absent, 1 = present
	Solid tumor / Hematological malignancy	0 = absent, 1 = present
	Polypharmacy	≥5 drugs = 1
**Preoperative variables**	Fracture type	Intertrochanteric / Subtrochanteric / Femoral neck
	Fixation type	DHS/DCS, PFN/IMN, Cannulated screws, Arthroplasty
	ASA score	1–4
	Type of anesthesia	General / Spinal / Combined spinal-epidural
	Time from fracture to surgery (TFS)	Continuous (days)
**Preoperative labs**	Hemoglobin (Hgb)	Continuous; Anemia if <12 g/dL
	Albumin (Alb)	Continuous; Hypoalbuminemia if <3.5 g/dL
	White blood cell (WBC)	Continuous; Low if <3,460/μL
	Platelets (Plt)	Continuous; Low if <172,000/μL
	CRP	Continuous; Elevated if >5 mg/L
**Postoperative data**	Postoperative anticoagulant use	0 = absent, 1 = present
	Length of ICU stay	Continuous (days)
	Total hospital stay	Continuous (days)
**Complications**	Anemia (postop)	0 = absent, 1 = present
	Hypoalbuminemia (postop)	0 = absent, 1 = present
	Electrolyte imbalance	0 = absent, 1 = present
	Pneumonia	0 = absent, 1 = present
	Heart failure	0 = absent, 1 = present

	Deep vein thrombosis / Pulmonary embolism	0 = absent, 1 = present
	Urinary infection	0 = absent, 1 = present
	Delirium	0 = absent, 1 = present
	Liver dysfunction	0 = absent, 1 = present
	Mortality	0 = survived, 1 = died

Supplementary Table S3 provides detailed information on data preprocessing and variable definitions to ensure reproducibility and transparency, in line with TRIPOD guidelines.

Suplemantary Table S3. Data preprocessing and variable definitions

**Step**	**Description**	**Details / Definitions**
**1. Missing data**	Screening for completeness	No missing values were present in any of the demographic, clinical, or laboratory variables.
**2. Variable coding**	Categorical variables	Binary coded (0 = absent, 1 = present). Examples: comorbidities, fracture type, anesthesia type.
**3. Continuous variables**	Initial analysis	Continuous laboratory variables were first analyzed as continuous measures in univariate analyses.
**4. Dichotomization**	Reference cut-offs applied for clinical interpretability	WBC <3,460/µL = low; Hgb <12.1 g/dL = anemia; Alb <3.5 g/dL = hypoalbuminemia; Plt <172,000/µL = low; CRP >5 mg/L = elevated.
**5. Outcomes**	Primary endpoint	Extended length of stay (LOS), defined as >7 days from surgery to hospital discharge (including ICU stay).
**6. Feature selection**	LASSO regression with 10-fold cross-validation	Optimal penalty $\lambda = 0.0249$ (λmin). 14 predictors retained.
**7. Modeling**	Algorithms applied	Logistic regression, Random Forest, XGBoost, Decision Tree.
**8. Validation**	Training/test split	Dataset randomly split 80/20. Hyperparameter tuning with 10-fold CV on training set. Final performance reported on independent 20% test set.
**9. Normalization**	Requirement check	Not required, as all predictors were binary or categorized by thresholds.
**10. Interpretability**	Post-model analysis	SHAP (Shapley Additive exPlanations) applied to XGBoost model for feature contribution analysis.

Supplementary Table S4. Full coefficients of the multivariate logistic regression model for predicting extended hospital stay

**Predictor Variable**	**β Coefficient**	**Interpretation**
WBC (Low)	1.78	Increased risk
Hemoglobin (Hgb)	0.04	Increased risk (per g/dL)
Time from fracture to surgery	0.55	Increased risk (per day)
Intertrochanteric fracture	-0.03	Decreased risk
Subtrochanteric fracture	0.48	Increased risk
Peptic ulcer disease	-1.06	Decreased risk
Liver disease	-1.81	Decreased risk
Solid tumor	0.24	Increased risk
Polypharmacy	0.33	Increased risk
Total hip arthroplasty	0.18	Increased risk
DHS or DCS fixation	-0.41	Decreased risk
PFN or IMN fixation	-0.74	Decreased risk
Cannulated screws	1.08	Increased risk
Spinal anesthesia	-0.73	Decreased risk
Combined spinal–epidural	0.34	Increased risk
ASA class 2	-0.97	Decreased risk
ASA class 3	-0.46	Decreased risk
ASA class 4	-0.28	Decreased risk
Anemia	0.47	Increased risk
Hypoalbuminemia	0.34	Increased risk
Electrolyte imbalance	0.66	Increased risk
Pneumonia	1.19	Increased risk
